# Soft Tissue Management on Pontic and Implant Sites Before Implants Insertion

**DOI:** 10.7759/cureus.24621

**Published:** 2022-04-30

**Authors:** Carlos A Jurado, Chin-Chuan Fu, Luis G Guzman, Jose Villalobos-Tinoco, Akimasa Tsujimoto

**Affiliations:** 1 Prosthodontics, Texas Tech University Health Sciences Center, Woody L. Hunt School of Dental Medicine, El Paso, USA; 2 Restorative Dentistry, University of Alabama School of Dentistry, Birmingham, USA; 3 Periodontics, University of Alabama School of Dentistry, Birmingham, USA; 4 Specialty Program in Periodontics, Faculty of Dentistry - National University of Rosario, Rosario, ARG; 5 Operative Dentistry, University of Iowa College of Dentistry and Dental Clinics, Iowa City, USA

**Keywords:** implant restorations, ceramics, zirconia restorations, esthetic dentistry, oral implantology

## Abstract

Complex implant therapy can include methods requiring several phases of treatment, and they are usually referred to as one-stage and two-stage approaches. The reasons for the staged approach include the extraction of non-restorable teeth. Such a treatment approach may offer a fixed provisional prosthesis during implant osseointegration that enables the patient to avoid removable prostheses. However, this case aims to demonstrate how to manage the soft tissue in the pontic region prior to immediate implant placement.

A 45- years old female patient presented with non-restorable teeth from the maxillary right lateral incisor to the left lateral incisor were removed, followed by socket preservation and fixed provisional restoration from right maxillary canine to left canine. Soft tissue was contoured to achieve ovate shape by first with a tooth-supported provisional restoration from the maxillary left canine to the right canine and then by re-shaping with carbide and diamond burs; after the tissue obtained the desired architecture, implants were inserted on sites of the maxillary right lateral incisor and left central lateral incisor without immediate loading, but the same provisional fixed restoration maintained the previously contour tissue. Once implant osseointegration was achieved, screw-retained provisional restoration was placed, followed by the definitive fixed implant restoration. Because the soft tissue was previously contoured, the screw-retained implant provisional restorations maintained the tissue architecture.

These initial contouring procedures provided a more predictable outcome for the final tissue contour after implants were inserted. The final re-shaping with the implant screw-retained provisional restorations was minimum, and prostheses followed the previously provided tissue architecture. Before the endosteal implants are inserted, soft-tissue contouring prior to implant placement may provide a more predictable outcome of the final tissue architecture for pontic and implant areas. The patient and clinician can evaluate the success and limitations of tissue contouring prior to implant placement. It may also shorten the time required for tissue contouring with provisional implant restorations.

## Introduction

The esthetic management and preservation of tissue stability in the area surrounding implant abutments have been important topics for investigation and discussion [1,2]. The esthetics of implants replacing the anterior maxillary teeth are particularly challenging. Patients are very conscious of this area and have high demands, especially for harmony between the implants and the soft tissue [3-5]. Soft tissue management to achieve this has conventionally been performed after implant placement using implant-supported provisional restorations [6].

However, when this is achieved through surgical shaping and pressure from the implant-supported provisional restorations, the soft tissues surrounding the implant and other pontic areas conform to the desired shape. However, inflammation is a common complication, especially in multiple teeth restorations [7,8]. This inflammation has several causes, including surgery and pressure, which often lead to inflammation directly. While managing the inflamed soft tissues is in progress, implants are not fully adapted to one another, leaving space where food particles can be retained and plaque develops, causing further inflammation [9]. This inflammation could reduce the stability of osseointegration and lead to peri-implantitis. This case study demonstrates a technique for managing the soft tissue before implant placement, which should avoid these problems. This case report demonstrates the soft tissue contouring and the clinical outcome after implant loading.

## Case presentation

**Figure 1:**
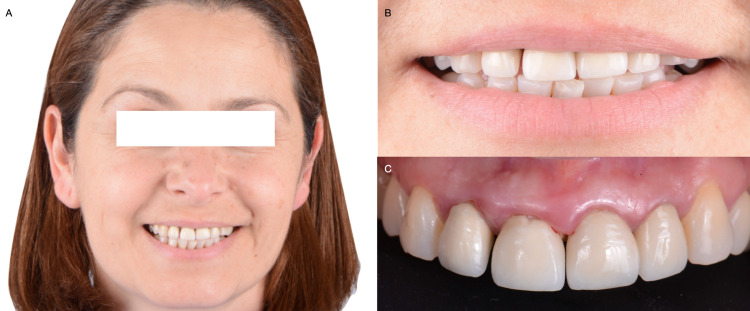
A) Initial face smiling, B) Initial smile, and C) Initial intra-oral situation.

A 45-years-old female patient presented to the clinic with the chief complaint, "My front crowns are loose, and they come off sometimes". The patient reported that she had crowns made of porcelain fused to metal placed on teeth from the maxillary right lateral incisor to the left incisor fifteen years ago (Figures 1). These crowns became loose and came off sometimes, which she previously cemented back on with an over-the-counter remedy from the pharmacy. The interdental papilla was reduced in height, forming a black triangle between anterior teeth.

**Figure 2:**
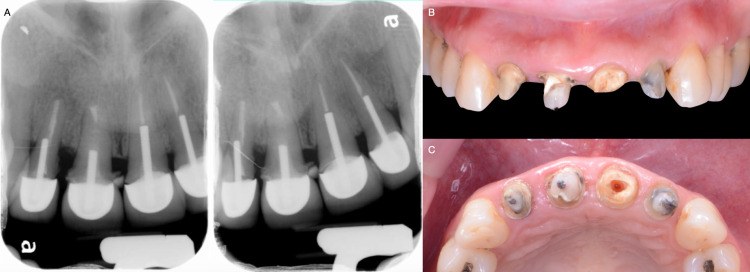
A) Initial radiographs and B) Clinical evaluation after crown removal.

After a detailed clinical evaluation, the maxillary right lateral incisor, central incisor, and maxillary left lateral incisor were diagnosed with mobility grade II. Incisal wear was found on the maxillary right and left canines. Radiographic evaluation showed the old crowns and metal posts on the maxillary left lateral incisor, central incisor, and right lateral incisor (Figure 2A). The patient had high esthetic demands and showed interest in having fixed all-ceramic restorations from the right maxillary canine to the left canine. Fortunately, the patient had a low smile line, and gingival inflammation and disharmony were not showing while smiling. The patient was informed of the need to remove the old crowns to re-assess the teeth' clinical situation and agreed to the procedure.

The existing restorations of the maxillary incisors were removed, and secondary caries were found in all teeth. A fractured core was observed on the maxillary left central incisor with grade II mobility on all teeth (Figures 2B, 2C). Therefore, these teeth were deemed hopeless. The patient was presented with the option of having two implants placed to support a fixed prosthesis from the maxillary left lateral incisor to maxillary right lateral incisors and single restorations on the maxillary right and left canines. The patient approved the plan, and the treatment was initiated.

**Figure 3:**
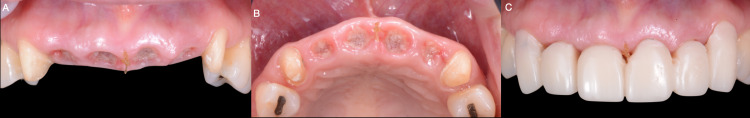
A) Tooth extractions, B) Suturing, and C) Provisional restoration.

A milled provisional restoration was fabricated based on pre diagnostic wax-up of the patient. The maxillary canines were prepared, and the patient was sent for teeth extractions. The maxillary central and lateral incisors were extracted. Particulate cortico-cancellous allograft bone (Cortical/Cancellous Chips, AlloSource Headquarters, Centennial, CO, USA) with collagen dressing (Puracol, Collagen Wound Dressing, Medline Industries Inc, Northfield, IL, USA) and resorbable sutures (Polysyn FA, Surgical Specialties Corporation, Wyomissing, PA, USA) was placed to achieve complete socket seal (Figures 3A, 3B). The milled provisional restorations, made of polymethyl methacrylate, were cemented with an ovate pontic shape in the extraction sites without interfering with the sutures. (Figure 3C) The initial provisional restorations applied light pressure and included a space between the soft tissue and the provisional restoration, enabling the patient to clean underneath the pontic and connector areas.

The patient returned two weeks later, and the provisional restorations were removed. The pontic units were built-up using self-curing acrylic resin (Jet Tooth Shade, Lang Dental, Wheeling, IL, USA) to establish contact under slight pressure and maintain the developed ovate soft tissue contour. The interproximal areas between the pontic units were opened with a disc (Acrylic Temporization System, Brasseler USA Dental, Savannah, GA. USA) to provide space for the papilla tissue. The patient again returned two weeks later, and the same procedure was performed.

**Figure 4:**
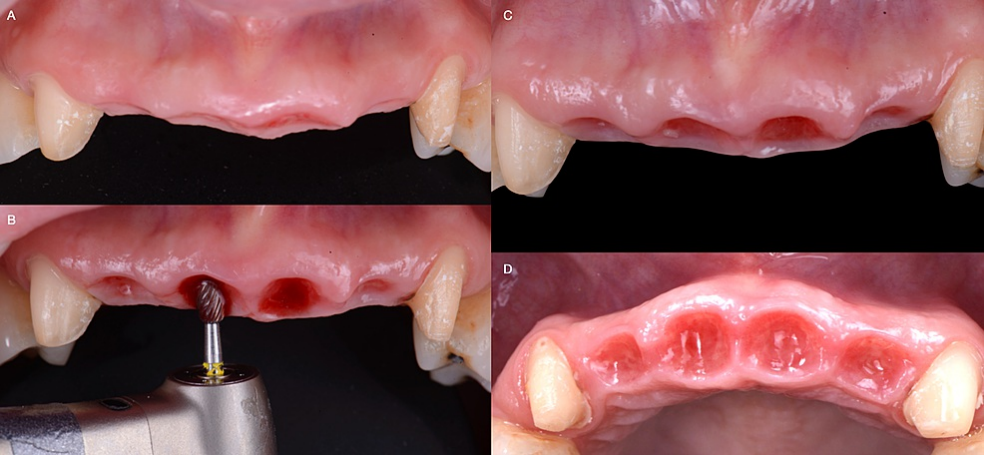
After two months of teeth extractions: A) Initial contour, B) Contouring soft tissue, C) Contoured tissue frontal view, and D) Contoured tissue occlusal view.

**Figure 5:**
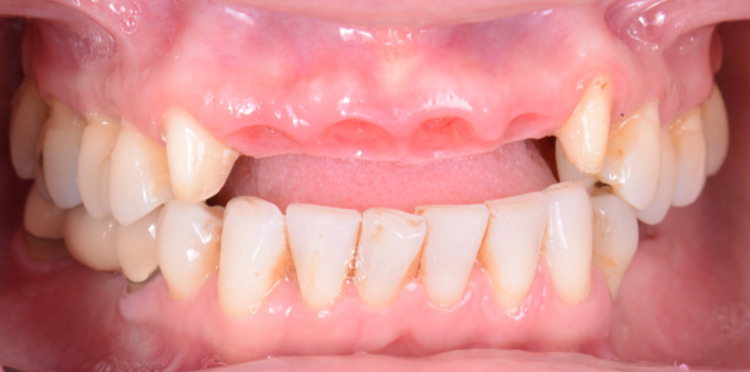
Final architecture of the soft tissue.

The patient was seen again for follow-up two months later, and the same procedure was performed. The thickness of the soft tissue was measured with a periodontal probe. (Williams Color-coded single end probe, Hu-Friedy Mfg. Co., LLC. Chicago, IL, USA) Fortunately, there was adequate keratinized tissue along with the maxillary labial site of the anterior teeth region. Carbide and diamond football burs (Medium Football bur, Brasseler USA Dental, Savannah, GA, USA) were used to improve the architecture of the pontic sites and prevent black triangles (Figures 4A, 4B). The provisional restoration was built-up again using acrylic resin material (Acrylic Temporization System, Brasseler USA Dental, Savannah, GA, USA) to match the contour provided by the football burs (Figure 4B, 4C). Two weeks later, the provisional restorations were removed to evaluate the final contour of the soft tissue (Figure 5).

**Figure 6:**
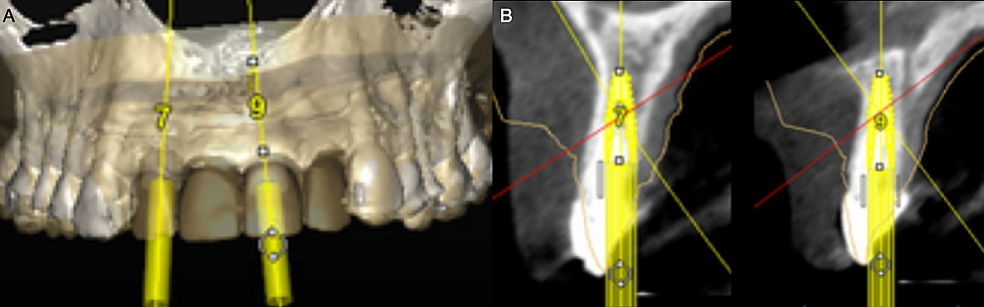
A) Frontal view of the digital implant planning. B) Interproximal view of the implant planning.

**Figure 7:**
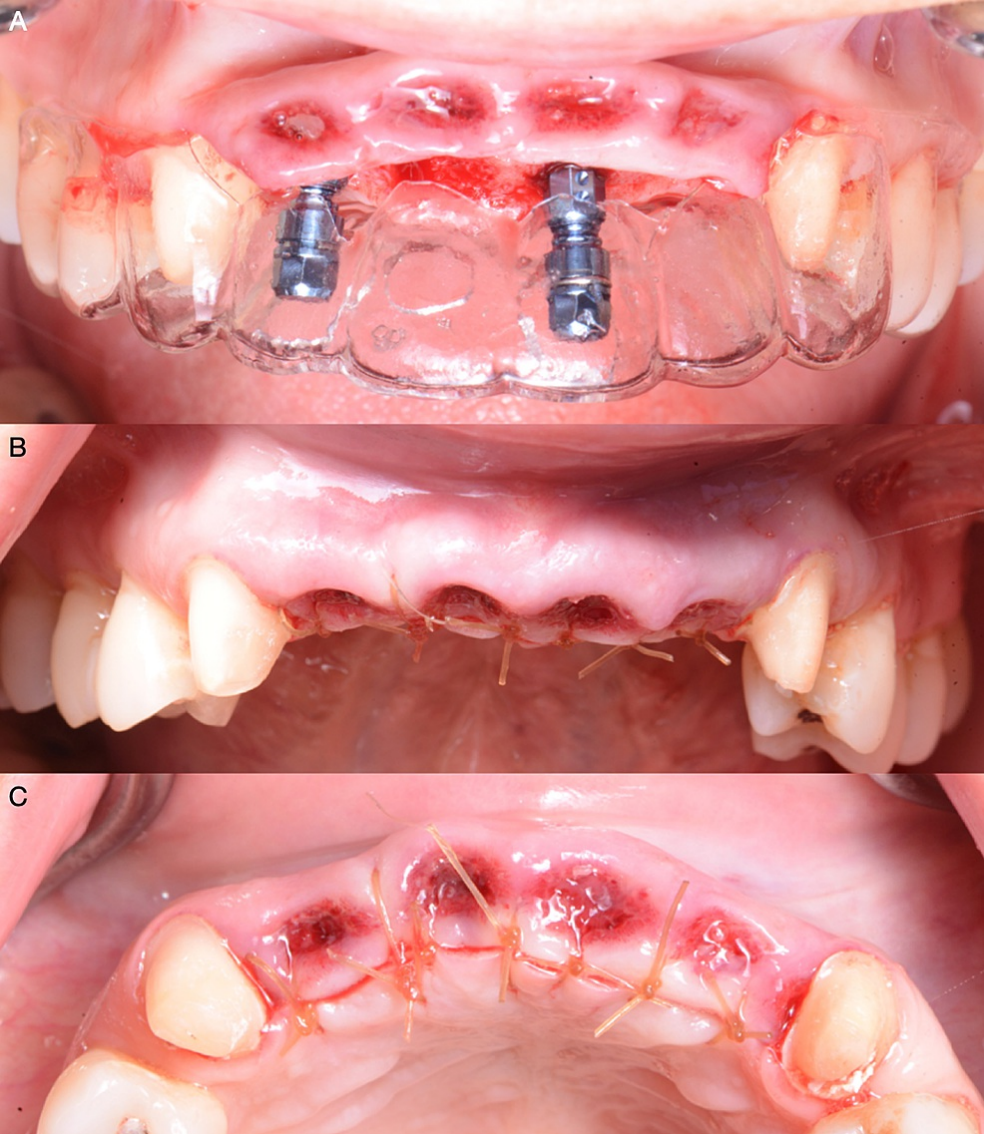
A) Implant placement, B) Suturing frontal view, and C) Suturing occlusal view.

The implant was planned using implant software after the soft tissue's desired shape was achieved (SimPlant, Dentsply Sirona Implants Inc, York, PA, USA) (Figure 6). Two implants at the maxillary right lateral incisor and maxillary left central incisor sites were planned to support a dental prosthesis from the maxillary left lateral incisor to the right lateral incisor. These sites were chosen based on the condition and thickness of the bone in the incisor region. Two weeks after the soft tissue contoured, the implant surgery was performed with a palatally-oriented crestal incision and bilateral sulcular incisions on the canines to reflect a full mucoperiosteal flap. The incision lines for the surgical flap were placed to avoid damaging the soft tissue line created during the preparation. Two bone-level implants of size 4.1mm (BLT RC, Straumann Group, Basel, Switzerland) were inserted (Figure 7A). For primary soft tissue closure, a simple interrupted suture technique (Polysyn FA, Surgical Specialties Corporation, Wyomissing, PA, USA) was used (Figure 7B, 7C). The implants were not loaded, and the existing fixed provisional restoration was cemented back onto the canines. During the two months of osseointegration, the provisional restorations maintained the soft tissue architecture that had been previously obtained.

**Figure 8:**
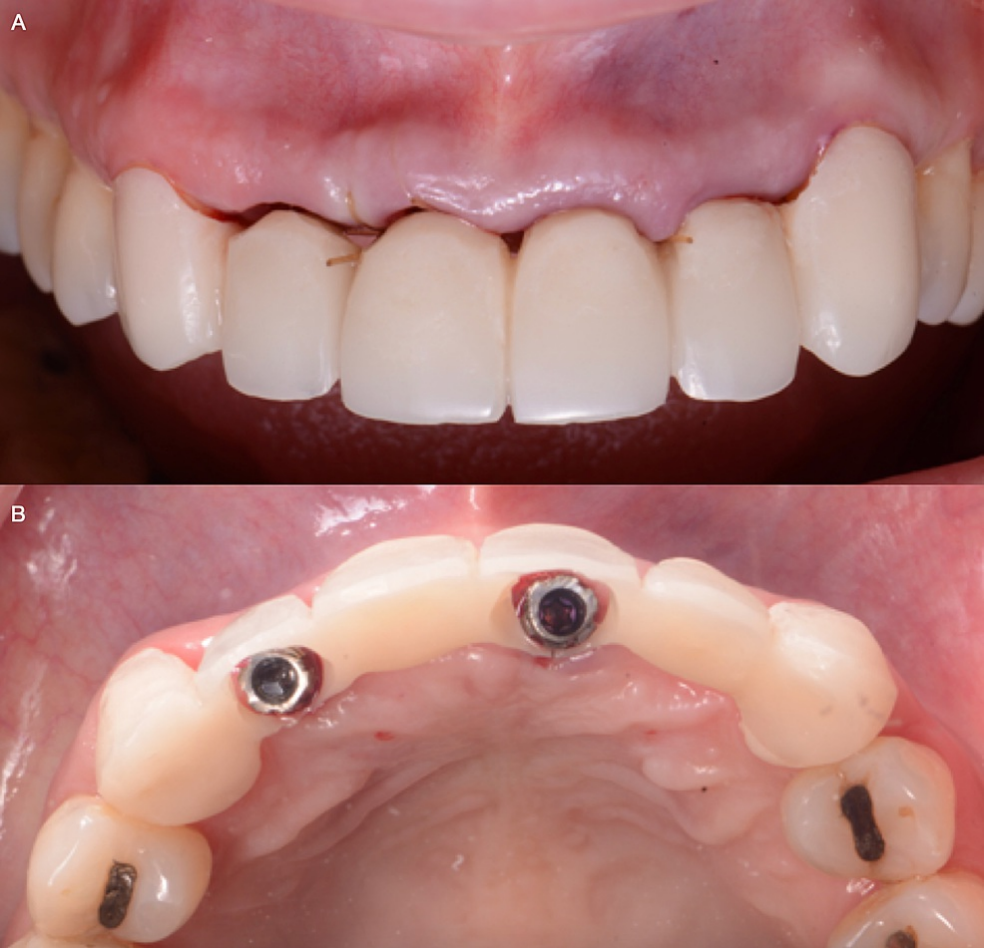
A) Screw retained implant provisional restoration frontal view, and B) Screw retained provisional restoration occlusal view.

**Figure 9:**
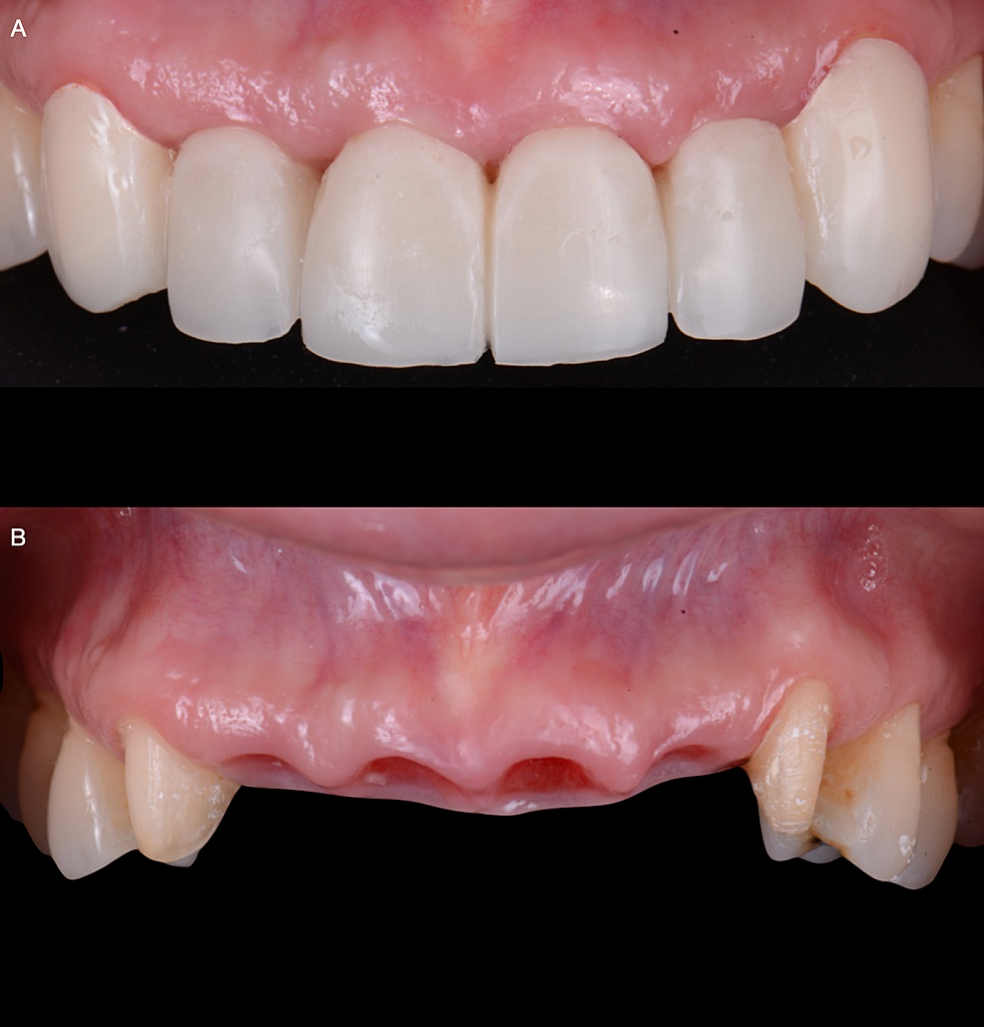
A) Screw retained provisional restoration frontal view, and B) Final tissue soft tissue architecture.

**Figure 10:**
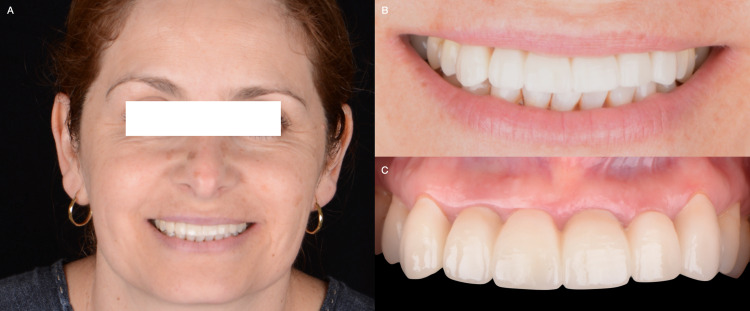
A) Final face smiling, B) Final smile, and C) Final intra-oral.

After four months, the pontic sites of the maxillary right lateral incisor and left central incisor was hollowed with acrylic carbide burs (Acrylic Temporization System, Brasseler, Savannah, GA, USA) in order to accept the temporary cylinders (Cylinder RC, Straumann Group, Basel Switzerland) engaged with the implants (Figures 8). The final restorations were screw-retained porcelain fused to zirconia with zirconia abutments fixed to titanium bases to replace the incisors and single porcelain fused to zirconia crowns on the canines (Figures 9). The new screw-retained provisional restorations maintained the same tissue contour, and three weeks later, a final impression was made (Fig 10).

Final restorations were tried-in for clinical and radiographic assessment, and patient satisfaction was achieved. Final implant restoration was placed and torqued according to the manufacturer's instructions, while single crowns on the maxillary right and left canines were cemented (RelyX Luting 2 Cement, 3M Espe, Saint Paul, MN, USA). Occlusion was checked and adjusted as necessary. The patient was satisfied with the outcome (Figures 10). A night guard was provided to protect the dentition and final restorations.

## Discussion

This technical report introduced a different approach for soft tissue contouring for implants in the esthetic zone. Traditionally, peri-implant tissue is contoured after the insertion of the implant placement. However, before the endosteal implants are inserted, soft-tissue contouring prior to implant placement may provide a more predictable outcome in the final tissue architecture for pontic and implant areas. The patient and clinician can evaluate success and limitations prior to implant placement, and it may also shorten the time required for tissue contouring with provisional implant restorations.

Optimal esthetics for implant therapy can be achieved by proper 3-dimensional planning, ideal implant depth with adjacent teeth, and peri-implant soft tissue molded by the provisional prosthesis [10,11]. Immediate implant placement with provisional restoration is a standard procedure. The goal is to establish an ideal emergence profile with maximum tissue volume, preserving mid-facial gingiva and enhancing patient comfort and acceptability [12,13]. This then serves as a guide for designing and fabricating the final restoration [14]. However, the present report has shown that all these goals could be achieved on pontic and implant sites before the implants are inserted.

After soft tissue is idealized, as described in this report, it would be possible to perform flapless implant placement. This is suitable for patients with sufficient keratinized gingival tissue and bone volume in the implant recipient site. It has been reported that the flapless implant placement approach minimizes post-operative peri-implant tissue loss and therefore reduces the difficulties of soft tissue management after the surgical intervention [15]. In addition, a flapless implant approach may cause less traumatic surgery, decrease operative time, provide faster post-surgical healing, allow for fewer complications after surgery, and provide more comfort to the patient [15,16]. However, implanting through the prepared soft tissue risks damaging the prepared areas, rendering the prior treatment meaningless. If a flap is used, a simple secondary operation to create a hole for the implant is sufficient, reducing the potential damage to the shaped soft tissue. This has further advantages in allowing a clear view of the surgical site and fully penetrating irrigation water to the osteotomy, thereby preventing thermal damage [17]. Although we chose to use open flap surgery in this case, the results were satisfactory. Nevertheless, it would be helpful to investigate the comparison of the contouring procedure with the burs and the provisional restorations for flapless implant placement to determine the best overall technique.

The flap design used in this case was based on two primary considerations. Conventional open-flap dental implant therapy cuts the soft tissue on a line passing through the center of the implant location. This design has some similarities to the papilla preservation flap procedure, and however, in this case, the incision was made around the edge of the gingival tissue, creating a single flap that lifted off the area. First, this avoided any damage to the shaped soft tissue that might result from an incision passing through that area. Second, moving the sutured area away from the implant reduced the risk of infection in the newly-placed implant while the incisions were healing. This may have contributed to the successful outcome. However, the choice of incision location is influenced by many factors, and this approach may not be suitable in some cases. In the end, this procedure provided ideal peri-implant tissue levels that gave the aesthetics desired.

Following this protocol, the clinician and patient can see the future final tissue contour in the pontic sites before implant placement. This will enable both sides to agree on an appropriate strategy to achieve the desired esthetics if there are shortcomings in the remaining soft tissue.

## Conclusions

Soft-tissue contouring for implant restorations may be challenging, however, contouring the tissue prior to implant placement may provide a more predictable outcome in the final tissue architecture. This sequence allows the patient and clinician to evaluate the success and limitations of tissue esthetics prior to implant placement.
